# ROCK 1 and 2 affect the spatial architecture of 3D spheroids derived from human corneal stromal fibroblasts in different manners

**DOI:** 10.1038/s41598-022-11407-1

**Published:** 2022-05-06

**Authors:** Yosuke Ida, Araya Umetsu, Masato Furuhashi, Megumi Watanabe, Yuri Tsugeno, Soma Suzuki, Fumihito Hikage, Hiroshi Ohguro

**Affiliations:** 1 Departments of Ophthalmology, Sapporo Medical University School of Medicine, Sapporo, Japan; 2 Cardiovascular, Renal and Metabolic Medicine, Sapporo Medical University School of Medicine, Sapporo, Japan

**Keywords:** Eye diseases, Pharmacology

## Abstract

The objective of the current study was to examine the roles of ROCK1 and 2 on the spatial architecture of human corneal stroma. We examined the effects of a pan-ROCK inhibitor (pan-ROCK-i), ripasudil, and a ROCK2 inhibitor (ROCK2-i), KD025 on the expression of genes that encode for ECM proteins including collagen (COL) 1, 4, 6, and fibronectin (FN), their regulators, a tissue inhibitor of metalloproteinase (TIMP) 1–4, matrix metalloproteinase (MMP) 2, 9 and 14, and ER stress-related factors of two- and three-dimensional (2D and 3D) cultures of human corneal stroma fibroblasts (HCSFs), and the physical properties of 3D HCSF spheroids. A gene expression analysis using ROCK-is indicated that KD025 (ROCK2 selective ROCK inhibitor) induced more significant changes than Rip (ripasudil, pan-ROCK inhibitor), suggesting that ROCK2 might be more extensively involved in the metabolism of ECM proteins and cell architectures of the 2D cultured HCSFs than ROCK1. In terms of the physical properties, size and stiffness of the 3D HCSFs spheroids, Rip caused a significant enlargement and this enhancement was concentration-dependent while KD025 also exerted a similar but less pronounced effect. In contrast, Rip and KD025 modulated physical stiffness differently, in that Rip caused a substantial decrease and KD025 caused an increase. Such diverse effects between Rip and KD025 were also observed for the gene expressions of ECM proteins, their regulators, and ER-stress related factors. The findings presented herein suggest that the ROCK1 and 2 influence the spatial architecture of 3D HCFS spheroids in different manners.

## Introduction

The cornea is the transparent outer layer of the human eye and is composed of three distinct layers, namely, the epithelium, the stroma, and the endothelium, which function for protection, transparency, and in maintaining optical properties^1^. Among them, the stroma layer accounts for approximately 90% of the thickness of the cornea. In the case where the corneal stroma is affected by the disease, injury, or scarring, this may lead to a serious and sometimes irreversible loss of transparency. In fact, corneal stromal pathogenesis continues to be one of the major causes of blindness^2^. Structurally, the corneal stroma is composed of regularly arranged layers of collagen fibers interspaced with keratocytes^3^. Therefore, this unique spatial architecture would be expected to be the most important factor in maintaining corneal transparency as well as repair and regeneration. These collective findings suggest that developing a suitable in vitro model for replicating the human corneal stroma would be highly desirable in terms of developing a better understanding of the pathogenesis as well as therapeutic strategies for the treatment of this condition. In fact, several studies related to 3D cell cultures^4^ and the regeneration^5^ of the corneal stroma have been reported.

Keratocytes are known to be involved in multiple functions including both the synthesis and the degradation of collagen and participate in the transformation to a myofibroblast phenotype during the wound healing process^6^. Since the natural regeneration of the corneal stromal is a relatively slow process, sometimes occurring over at least several years^7^, such a long turnover time may be a prerequisite for healthy stromal regeneration for which a lamellar structure is needed for corneal strength and transparency to be maintained. While in contrast, a short period of repair is often associated with the production of aberrant collagen by myofibroblasts, thus resulting in stromal haze and the deposition of permanent scar tissue^8^. Although keratocytes are usually relatively quiescent, those are activated to proliferative fibroblasts and then to contractile myofibroblasts during the wound healing process^9,10^. It is known that this transition of keratocytes to fibroblasts is associated with the increased formation of actin filaments and stress fiber assembly^9,11^. A previous study indicated that Rho-associated coiled-coil containing protein kinase (ROCK) signaling regulates actin filament assembly and is probably a key factor in the regulation of these keratocyte transitions^12^. It is well known that ROCK is involved in a variety of physiological functions, including chemotaxis, neural growth, smooth muscle contraction^13–15^ in addition to the assembly and organization of actomyosin filaments^16–18^. In fact, the ROCK subspecies, ROCK1 and 2 are widely distributed in tissues including ocular tissues^19^, and are also involved in the development of several types of disease including glaucoma, cataracts, retinopathy, and corneal dysfunction^20–23^. These collective findings suggest that ROCKs may be promising therapeutic targets for the treatment of these ROCK involved diseases. In fact, ripasudil, a pan-ROCK inhibitor (pan-ROCK-i) has been available as a new option of a hypotensive medication for patients with glaucoma and ocular hypertension^24^. Furthermore, a recent study reported that ROCK-is also facilitates wound healing of corneal epithelium^25^ as well as the corneal stroma^26^. However, as of this writing, the roles of each ROCK1 and 2 subspecies remain to be elucidated, especially in terms of corneal stromal pathophysiology. To study the pathophysiological roles of ROCK isoform on the corneal stromal cells, we used a ROCK2 selective inhibitor, KD025, in addition to pan-ROCK inhibitor, Rip.

Therefore, in the present study, to elucidate possible roles of ROCK1 and 2 on the spatial architecture of the corneal stroma, drug induced effects of the pan-ROCK-i, Rip and the ROCK2-i, KD025 toward (1) the gene expression of ECM proteins, their regulators, and ER-stress related factors of human corneal stromal (HCSFs) cells were examined using our recently developed three-dimension (3D) drop culture^27–31^ in addition to conventional two-dimension (2D) cultures, and (2) on the physical properties of the 3D HCSF spheroids.

## Materials and methods

The current study, which was conducted at the Sapporo Medical University Hospital, Japan, was approved by the institutional review board (IRB registration number 282-8) according to the tenets of the Declaration of Helsinki and national laws for the protection of personal data. Informed consent was obtained from all subjects who participated in this study. The gene expressions of ROCK 1 and ROCK 2 within 2D and 3D cultured human corneal stroma fibroblasts (HCSFs) were confirmed prior to the current study (Supplemental Fig. 1).

### Preparation of 2D cultured HCSFs and 3D HCSFs spheroid

Surgically obtained human corneal stromal cells from 2 patients with traumatic perforating injuries during their ocular evisceration were used for preparing the 2D and 3D HCSFs cultures described below. The demographic data for these 2 patients is shown in Supplementary Table 1. The protocol for the isolation of human corneal stroma fibroblasts was basically performed by the previously described method^4^ with minor modifications.

The HCSFs cells, obtained as above, were grown in 150 mm 2D culture dishes until reaching 90% confluence at 37 °C in growth medium A composed of HG-DMEM containing 10% FBS, 1% l-glutamine, 1% antibiotic–antimycotic and were maintained by changing the medium every other day. These 2D cultured HCSFs were further processed to prepare 3D spheroids using a hanging droplet spheroid three-dimension (3D) culture system, as described recently^27^. Briefly, 2D cultures HCSFs as above were collected after washing with phosphate buffered saline (PBS), and re-suspended in spheroid medium A which was composed of growth medium A supplemented with 0.25% methylcellulose (Methocel A4M) to facilitate stable 3D spheroid morphology. Approximately, 20,000 HCSFs in 28 μL of spheroid medium A were added to each well of the hanging drop culture plate (# HDP1385, Sigma-Aldrich) (Day 0). At Day 1, ROCK-i, Ripasudil (Rip) (a generous gift from the Kowa Company Ltd., Nagoya, Japan) or KD025 at different concentrations (0, 1, or 10 μM) was added to the spheroid medium A until Day 6. On each following day, 14 μL of the medium was replaced with14 μL of fresh medium. At Day 6, these 3D HCSFs spheroids under different conditions as above were collected and used in the analyses described below.

### Quantitative PCR

Total RNA was extracted from 2D cultured cells within a single well out of 12 wells of the culture dish or 16 spheroids using an RNeasy mini kit (Qiagen, Valencia, CA). Reverse transcription was performed with the SuperScript IV kit (Invitrogen) as per the manufacturer’s instructions. Respective gene expressions were quantified by real-time PCR with the Universal Taqman Master mix using a StepOnePlus instrument (Applied Biosystems/Thermo Fisher Scientific). The quantities of cDNA were normalized to the expression of the housekeeping gene 36B4 (*Rplp0*) and are shown as fold-change relative to the control. The sequences of primers and Taqman probes used are shown in Supplementary Table 1.

### Measurement of the physical properties, size, and stiffness of 3D spheroids

As described previously, the 3D spheroid configurations were observed by phase contrast (PC, Nikon ECLIPSE TS2; Tokyo, Japan), and the mean size of each 3D spheroid, as defined as their largest cross-sectional area (CSA), were calculated using the Image-J software version 1.51n (National Institutes of Health, Bethesda, MD)^27^.

The physical solidity of the 3D spheroids was measured using a micro-squeezer (MicroSquisher, CellScale, Waterloo, ON, Canada) equipped with a microscale compression system composed of a 406 μm diameter cantilever, as reported in a recent study^27^. A single spheroid was placed on a 3-mm × 3-mm plate and then compressed to a 50% deformation for a period of 20 s, as determined by a microscopic camera. The force required to produce a 50% strain was measured through the cantilever under several different osmotic pressures, and the data are expressed as force/displacement (μN/μm).

### Immunostaining of 3D spheroids

Immunostaining of the 3D HCSFs spheroids was carried out as described previously using a primary antibody (1:200 dilutions); a rabbit anti-collagen monoclonal antibody (collagen 1; # 600-401-103-0.1, collagen 4; # 600-401-106-0.1, or collagen 6; # 600-401-108-0.1, Rockland Immuno-chemicals Inc.) or a mouse anti-FN monoclonal antibody (# G0717, Santa Cruz Biotechnology), and the 2nd antibody (1: 500 dilutions) being goat Alexa Fluor 488 anti-rabbit IgG (# A-11070, Thermo Fischer Scientific) or goat Alexa Fluor 594 anti-mouse IgG (# A-11020, Thermo Fischer Scientific)^32^. Nuclear staining for F-actin has been accomplished with Alexa Fluor 594 phalloidin (# 20553, Funakoshi) and DAPI (# D523, Dojindo) at 1:1000 dilutions for 3 h at room temperature. The fluorescence intensity of each ECM was measured using a Nikon A1 confocal microscope (Tokyo, Japan) and quantified using the Image J software version 2.0.0 (NIH, Bethesda, MD) as described in a previous report^27^. In brief, for the fluorescence intensity per unit area of spheroid, 2D images of each spheroid obtained by serial z-axis imaging (2.0-μm interval) at a 65-μm range from the surface of the organoid were collected and converted to a Z-stack image. The maximum intensity within the Z-stack image was evaluated and the signal intensity per spheroid was expressed as intensity/surface area during the z-plane using the NIS-Elements 4.0 software.

### Statistical analyses

All statistical analyses were performed using Graph Pad Prism 9 (GraphPad Software, San Diego, CA). A two-tailed Student’s t-test was used to calculate statistical significance with a confidence level greater than 95% to compare two mean values. A grouped analysis with two-way variance (ANOVA) followed by a Tukey’s multiple comparison test was performed to analyze the difference in groups. Data are presented as the arithmetic mean ± standard error of the mean (SEM).

## Results

In the present study, to evaluate the nature of the roles played by ROCK1 and 2 on the HCSFs, the effects of ROCK-is, the pan-ROCK inhibitor, ripasudil (Rip), and the ROCK2 selective inhibitor, KD025 on a spectrum of molecules that are related to cell architecture, such as ECM proteins, their regulators and major ER stress related factors were investigated by qPCR analyses using 2D and 3D cultured HCSFs. In advance to the present study below, we verily the validity of use these cells, we examined the gene expression of *keratocan* (*KERA*)^33^, and *αSMA*^34^, which are specific markers for corneal stromal cells and fibroblast, respectively (Supplemental Fig. 2). The results indicate that keratocan and αSMA were negatively and positively expressed, respectively. However, previous study indicated that keratocan is not always detected within the corneal stromal cells and in fact, no keratocyte markers including keratocan within the fibroblastic monoculture like our present preparation, even though those was from corneal stromal cells^33^. In addition, in terms of the 3D HCSF spheroid, we confirmed that HCSF cells aligned properly and concentrically within the 3D spheroid without any evidence of unhealthy conditions such as necrosis and apoptosis (Supplemental Fig. 3).

### Effects of ROCK-is, the pan-ROCK inhibitor, ripasudil (Rip), and the ROCK2 selective inhibitor, KD025 on the gene expression of ECM proteins, their modulators, and major ER stress related factors on 2D cultured HCSFs

**Figure 1:**
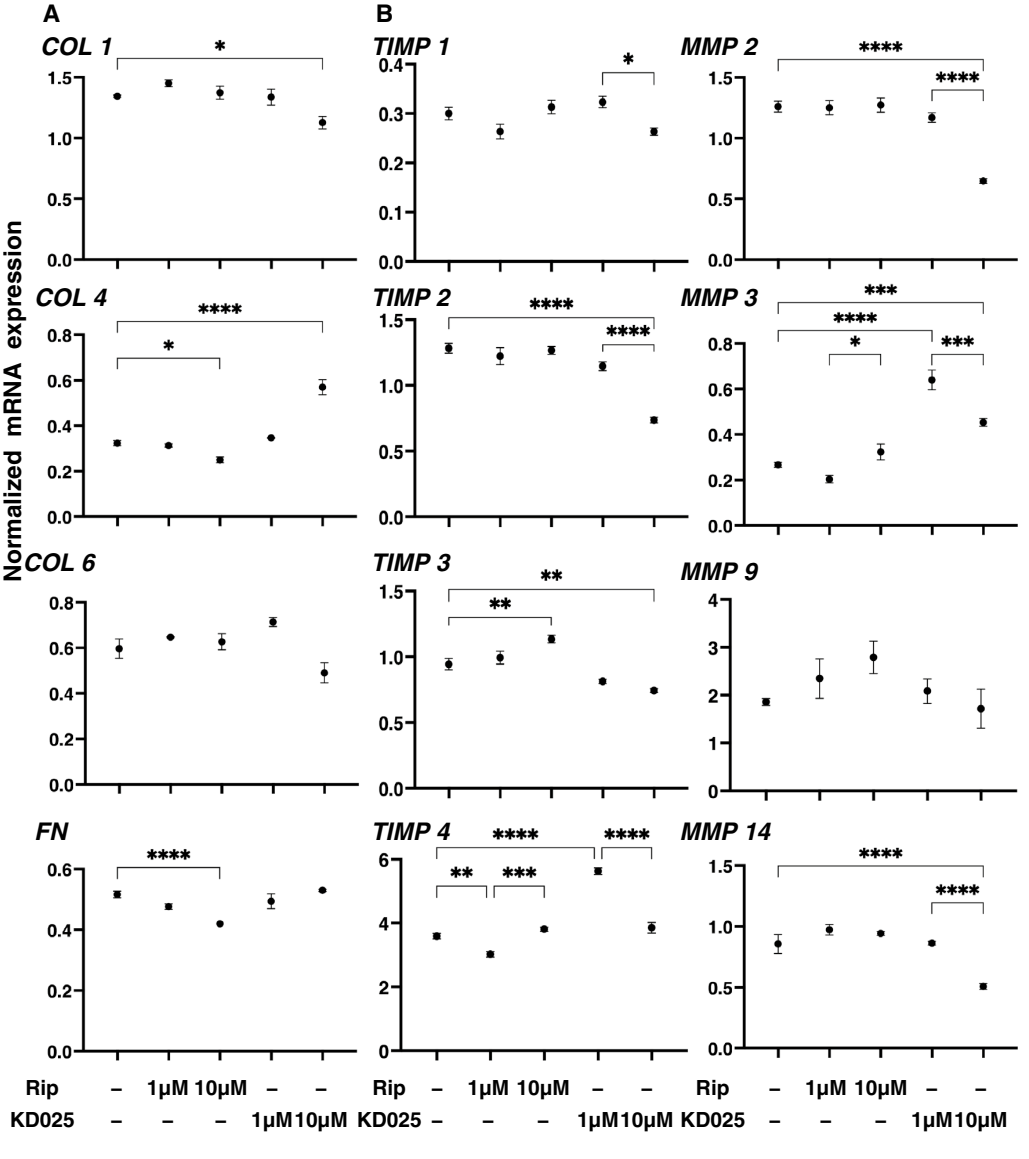
mRNA expression of ECM proteins, and their modulators, TIMPs (1–4) and MMPs (2, 3, 9, 14) in 2D cultured human corneal stroma fibroblasts (HCSFs). In the absence or presence of 1 μM or 10 μM ROCK-is, ripasudil (Rip), or KD025, 2D cultures of HCSFs at Day 6 were subjected to qPCR analyses to estimate the mRNA expression of ECM proteins including those for *COL1*, *COL4*, *COL6,* and *FN,* and their modulators, TIMPs (1–4) and MMPs (2, 3, 9, 14). All experiments were performed in duplicate using fresh preparations (n = 5, each). Data are presented as the arithmetic mean ± standard error of the mean (SEM). *P < 0.05, **P < 0.01, ***P < 0.005, ****P < 0.001 (ANOVA followed by a Tukey’s multiple comparison test).

**Figure 2:**
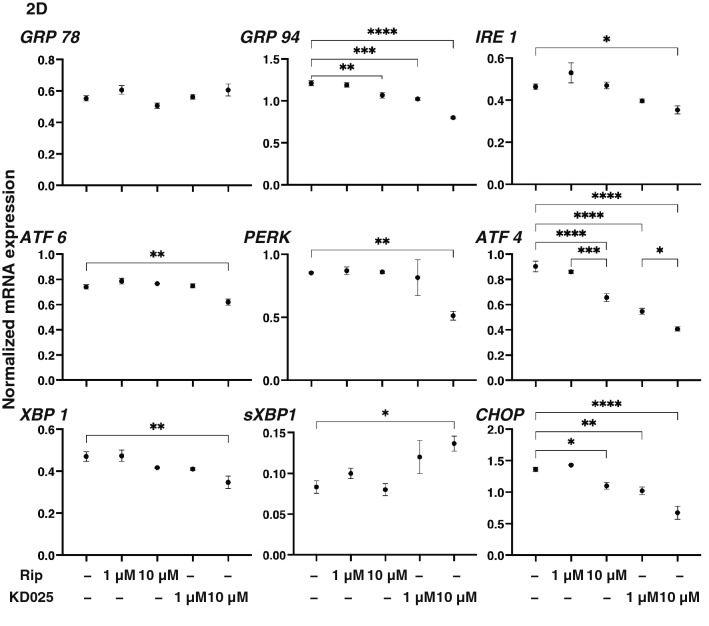
Effects of ROCK-is on the mRNA expression of major ER stress related genes in 2D cultured human corneal stromal fibroblasts (HCSFs). In the absence or presence of 1 μM or 10 μM ROCK-is, ripasudil (Rip) or KD025, 2D cultures HCSFs at Day 6 were subjected to qPCR analyses to estimate the expression of mRNA of major ER stress related genes of three master regulators, i.e., the protein kinase RNA-like endoplasmic reticulum kinase (PERK), activating transcription factor 6 (ATF6) and the inositol-requiring enzyme 1 (IRE1), and their downstream factors including the glucose regulator protein (GRP)78, GRP94, the X-box binding protein-1 (XBP1), spliced XBP1 (sXBP1) and CCAAT/enhancer-binding protein homologous protein (CHOP). All experiments were performed in duplicate using fresh preparations (n = 5, each condition). Data are presented as the arithmetic mean ± standard error of the mean (SEM). *P < 0.05, **P < 0.01, ***P < 0.005, ****P < 0.001 (ANOVA followed by a Tukey’s multiple comparison test).

As shown in Fig. 1A, 10 μM Rip or KD025 induced significant down-regulation in *COL4* and *FN*, the substantial down-regulation of *COL1,* and the up-regulation of *COL4*, respectively among the four major ECM proteins. In addition, Rip or KD025 also induced the gene expression of modulators of ECM proteins, the tissue inhibitor of metalloproteinases (TIMPs) and the matrix metalloproteinases (MMPs) (Fig. 1B) as well as major ER stress related factors (Fig. 2); (1) rip induced a significant up-regulation of TIMP3 (10 μM) and the down-regulation of TIMP4 (1 μM), GRP94 (10 μM), ATF4 (10 μM) and CHOP (10 μM), and (2) KD025 induced a significant up-regulation of TIMP4 (1 μM) and MMP3 (1 and 10 μM), and the down-regulation of TIMP2 (10 μM), TIMP3 (10 μM), TIMP14 (10 μM) and all ER stress related factors except GRP78 and sXBP1 (1 or/and 10 μM). Based on the above gene expression analysis using ROCK-is, ROCK2 appears to be more importantly involved in the metabolism of ECM proteins and the cell architectures of the 2D cultured HCSFs than ROCK1.

### Effects of ROCK-is, a pan-ROCK inhibitor, ripasudil (Rip), and the ROCK 2 selective inhibitor, KD025 on the physical properties and gene expression of several ECM proteins, their modulators, and ER-stress related genes in 3D HCSFs spheroids

**Figure 3:**
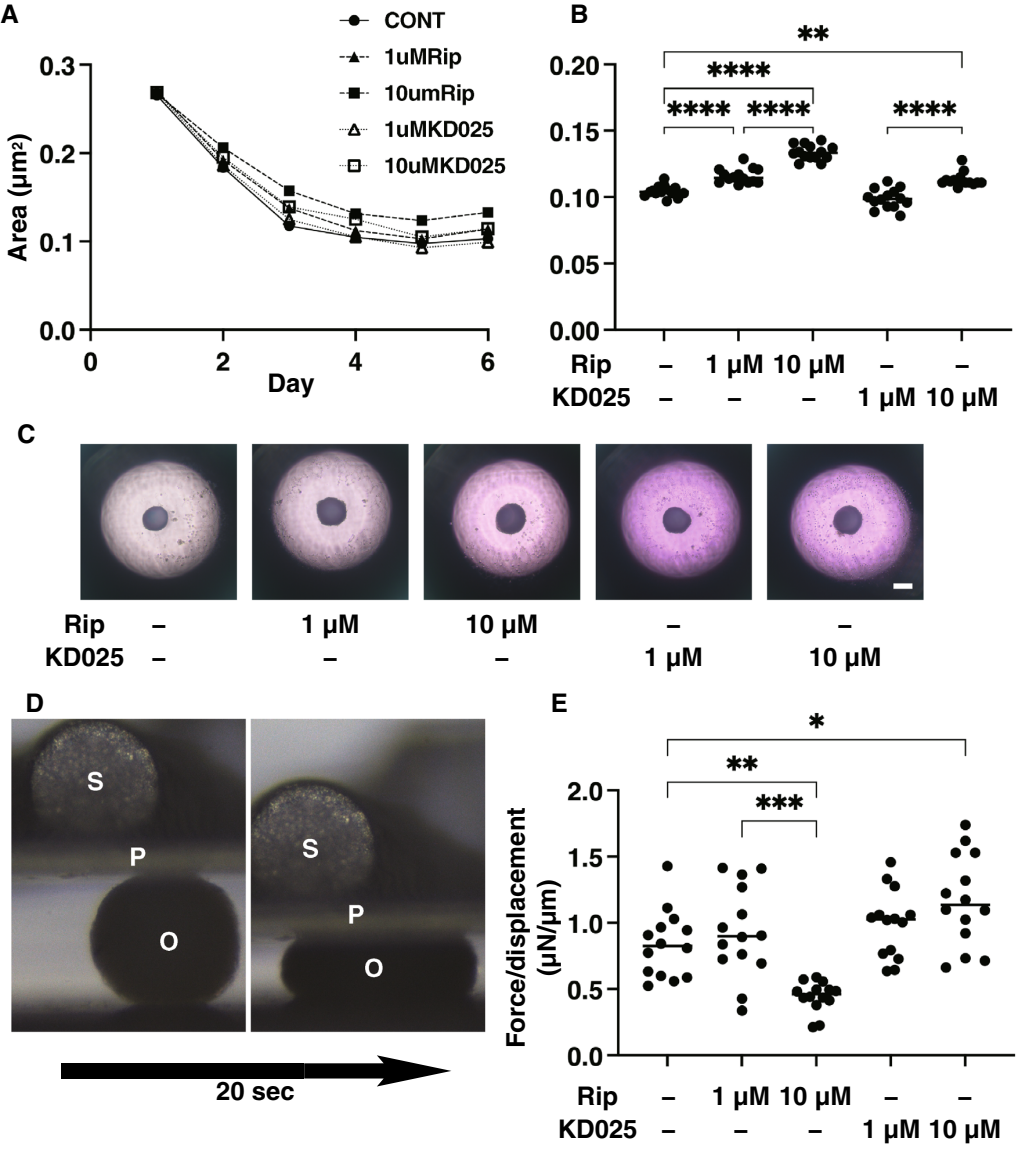
Effects of ROCK-is on physical properties, the mean sizes and stiffness of 3D spheroids of human corneal stroma fibroblasts (HCSFs) in the presence of ROCK inhibitors. In the absence or presence of 1 μM or 10 μM ROCK-is, ripasudil (Rip), or KD025, the mean sizes of 3D HCSFs spheroids were measured in triplicate using fresh preparations consisting of 16 organoids each during the 6-day culture period were plotted (**A**) and those at Day 6 were compared among the experimental groups (**B**), and phase contrast images are shown (**C**). Alternatively, using a micro-squeezer, the physical stiffness of a single living 3D HCSFs spheroid at Day 6 was measured during a 20 s period (**D**, O; 3D spheroid, P; compressing plate, S; pressure sensor) in the absence or presence of 1 μM or 10 μM ROCK-is, ripasudil (Rip) or KD025 (n = 15–20 freshly prepared 3D spheroids under each experimental condition). The force (μN) required to induce a 50% deformity was calculated and force/displacement (μN/μm) values were plotted in (**E**). Data are presented as the arithmetic mean ± standard error of the mean (SEM). *P < 0.05, **P < 0.01, ***P < 0.005, ****P < 0.001 (ANOVA followed by a Tukey’s multiple comparison test).

Next, to elucidate roles of ROCK1 and ROCK2 in the 3D corneal stromal architecture, the drug-induced effects of the ROCK-is, a pan-ROCK inhibitor, ripasudil (Rip), and the selective ROCK 2 inhibitor, KD025 on the physical properties of the 3D HCSFs spheroid were examined. As shown in Fig. 3A–C, uniform round-shaped spheroids of HCSFs grew into smaller matured forms during the 6-day culture, but their sizes were significantly modulated by Rip or KD025. That is, a significant enlargement was observed by Rip and this enlargement was concentration-dependent. KD025 also exerted a similar but less enlargement effect. In contrast, however, Rip and KD025 exerted different effects on the physical stiffness of the spheroids. That is, as shown in Fig. 3D,E, the physical stiffness of the 3D HCSF spheroids was substantially decreased by the addition of 10 μM Rip and was increased by KD025, respectively. These collective findings suggest that ROCK 1 induces the formation of smaller and stiffer spheroids, and ROCK2 induces the formation of softer 3D HCSF spheroids, respectively.

**Figure 4:**
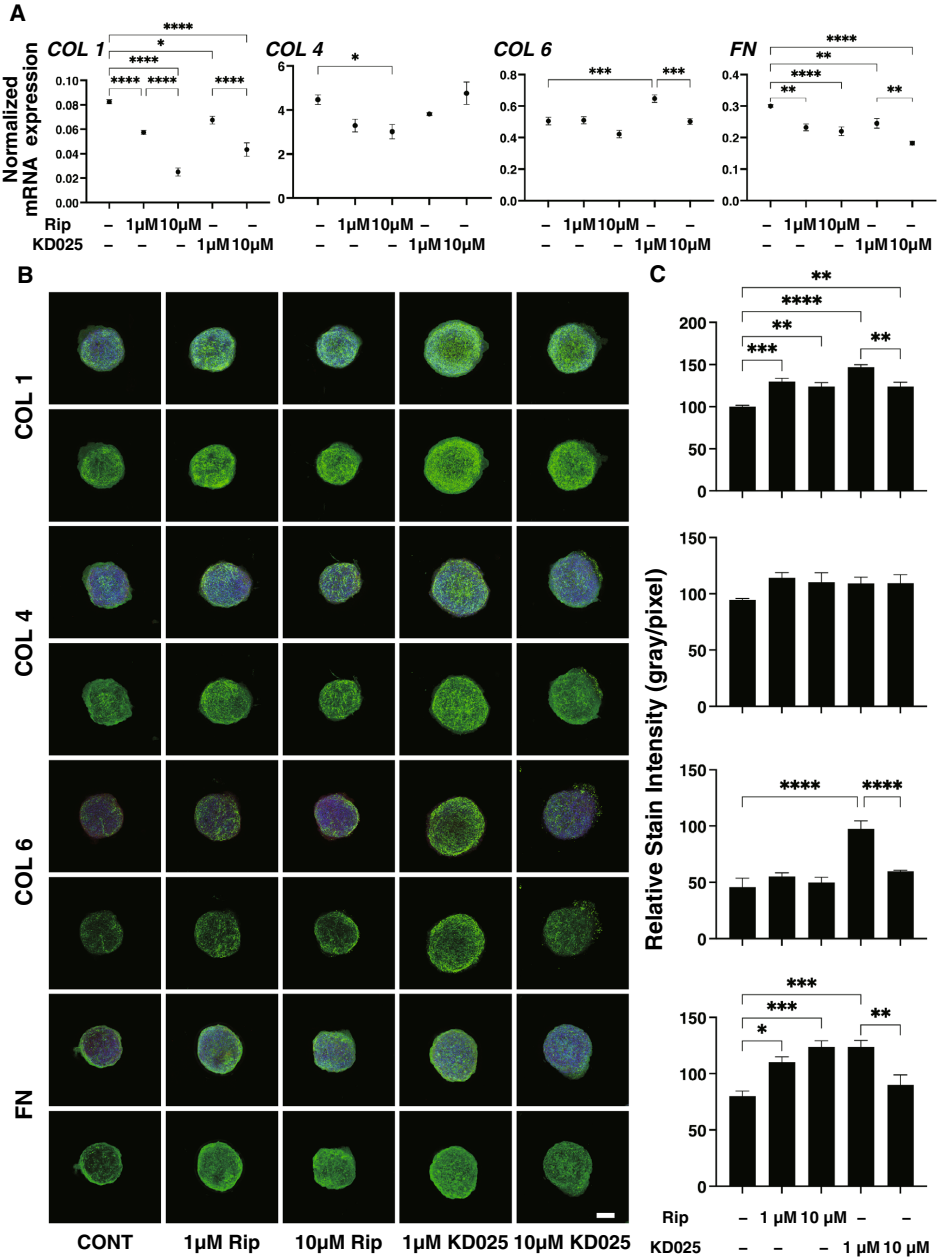
Effects of ROCK-is on the mRNA expression of ECMs in 3D spheroids of human corneal stroma fibroblasts (HCSFs) and representative immune-fluorescein images showing their expressions. In the absence or presence of 1 μM or 10 μM ROCK-is, ripasudil (Rip), or KD025, 3D HCSFs spheroids at Day 6 (n = 10–15, each) were subjected to qPCR analysis, and the mRNA expression of ECM molecules including *COL1*, *COL4*, *COL6,* and *FN* was estimated (**A**). Alternatively, those 3D spheroids (n = 4–8 each) were immune-stained with specific antibodies to ECMs including collagen 1 (COL 1), collagen 4 (COL 4), collagen 6 (COL6), or fibronectin (FN) designated by green, DAPI by blue, and phalloidin by red under above conditions. Representative confocal images are shown (**B**) (Scale bar: 100 μm). The staining intensities of the ECMs of the spheroids that were stained as above are plotted in (**C**). All experiments were performed in duplicate using fresh preparations. Data are presented as arithmetic means ± standard error of the mean (SEM). *P < 0.05, **P < 0.01, ***P < 0.005, ***P < 0.001 (ANOVA followed by a Tukey’s multiple comparison test).

To elucidate the underlying mechanism responsible for causing such ROCK-is induced effects on modulating the physical stiffness of the spheroids, the expressions of ECM molecules were evaluated (Fig. 4A). Results indicated that (1) the mRNA expression of ECM molecules, *COL 1* and *FN* were significantly down-regulated in the presence of Rip and KD025 and that this downregulation was concentration dependent, (2) the expression of *COL 4* was significantly down-regulated by 10 μM Rip and upregulated by 1 μM KD025, respectively. However, in contrast, immunocytochemistry demonstrated quite different results, that is, the relative stain intensities of COL1 and FN, and COL6 were significantly increased by both Rip and KD025, and KD025, respectively (Fig. 4B,C). Concerning our qPCR analysis using 3D spheroids, we previously confirmed that the data obtained by western blot analysis were consistent with the gene expression^27^. In fact, these discrepancies between levels of gene expression and immunolabeling were also often detected in our previous studies using 3T3-L1 cells^35^, human orbital fibroblasts^36^ and others^37^ because mRNA expression and immunolabeling may reflect the total expressions and the surface expressions of the target molecules.

**Figure 5:**
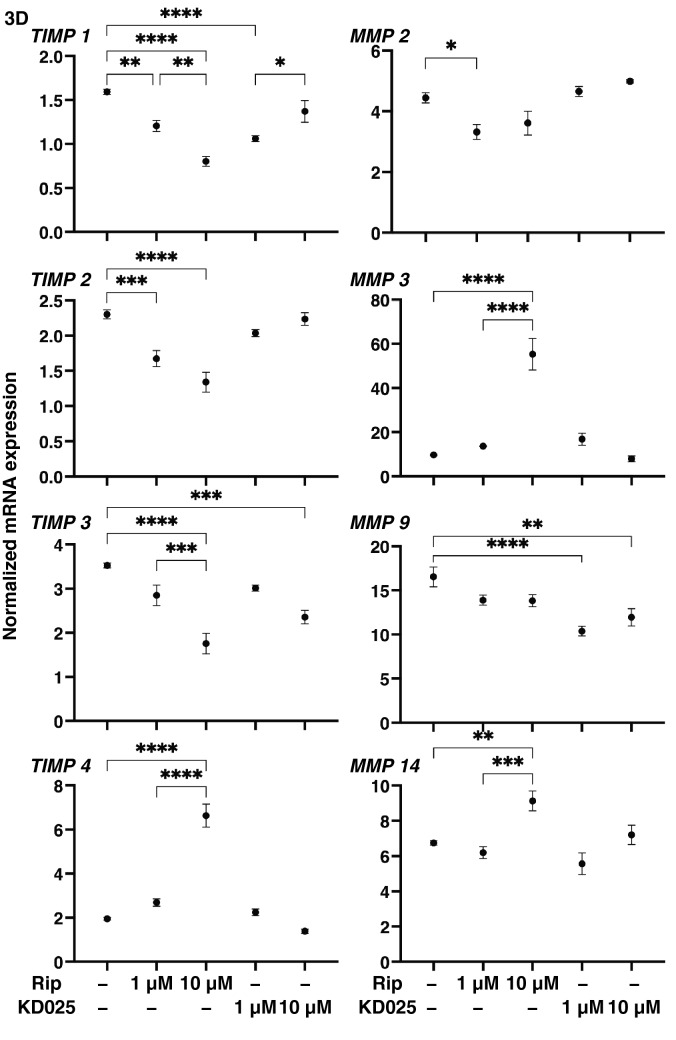
Effects of ROCK-is on the mRNA expression of TIMPs (1–4) and MMPs (2, 3, 9, 14) in 3D spheroids of human corneal stroma fibroblasts (HCSFs). In the absence or presence of 1 μM or 10 μM ROCK-is, ripasudil (Rip) or KD025, 3D HCSFs spheroids at Day 6 were subjected to qPCR analyses and the mRNA expression of TIMPs (1–4) and MMPs (2, 3, 9, 14) was estimated. All experiments were performed in duplicate using fresh preparations (n = 10–15, each). Data are presented as the arithmetic mean ± standard error of the mean (SEM). *P < 0.05, **P < 0.01, ***P < 0.005, ***P < 0.001 (ANOVA followed by a Tukey’s multiple comparison test).

**Figure 6:**
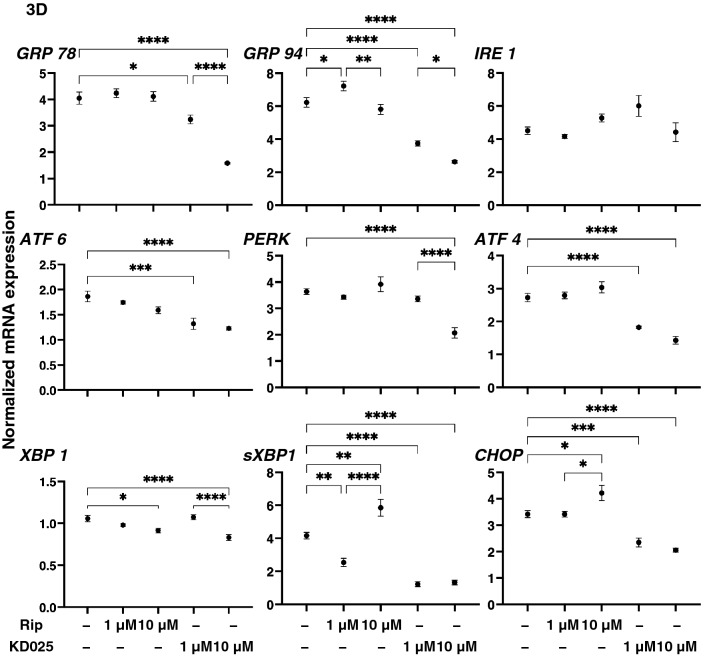
Effects of ROCK-is on the mRNA expression of major ER stress related genes in 3D human corneal stroma fibroblasts (HCSFs) spheroids. In the absence or presence of 1 μM or 10 μM ROCK-is, ripasudil (Rip) or KD025, 3D HCSFs spheroids at Day 6 were subjected to qPCR analyses and the expression of mRNA of major ER stress related genes of three master regulators, i.e., protein kinase RNA-like endoplasmic reticulum kinase (PERK), activating transcription factor 6 (ATF6) and the inositol-requiring enzyme 1 (IRE1), and their downstream factors including the glucose regulator protein (GRP)78, GRP94, the X-box binding protein-1 (XBP1), spliced XBP1 (sXBP1) and CCAAT/enhancer-binding protein homologous protein (CHOP) were estimated. All experiments were performed in duplicate using fresh preparations (n = 10–15, each condition). Data are presented as the arithmetic mean ± standard error of the mean (SEM). *P < 0.05, **P < 0.01, ***P < 0.005, ****P < 0.001 (ANOVA followed by a Tukey’s multiple comparison test).

To study this issue further, expressions of ECM modulators, TIMPs and MMPs (Fig. 5) and ER-stress related factors (Fig. 6) and found following observations; (1) the expression of *TIMP1-3 and TIPM4* was significantly down-regulated and that for *MMP3* was up-regulated by Rip in a concentration dependent manner, and (2) Rip induced the down-regulation of MMP2 and the up-regulation of MMP14, and KD025 induced the down-regulation of MMP9, respectively. Among these, the Rip and KD025-induced changes in *COL1* and *FN*, or *COL4* and *COL6* were quite similar to the changes in 3D spheroid sizes and stiffness caused by Rip or KD025. In addition, the changes in the expression of *TIMP1-3* and *MMP2* and *9* expressions were similar to the Rip induced changes of *COL1* and *FN*, and changes in the expression of TIMP1 2 and 4, MMP2 and 9 were also similar to the ROCK-is induced changes in the stiffness of the 3D HCSFs spheroids. Therefore, these collective data indicate that ROCK1 and 2 affect ECM proteins and their regulators in different manners, resulting in diverse effects on the physical properties of the 3D HCSFs spheroids. In support of these conclusions, the gene expressions of ER-stress related factors were also found to be affected by Rip and KD025 in different manners. As shown in Fig. 6, Rip modulated *GRP94, XBP1, sXBP1,* and *CHOP*, whereas KD025 induced the down-regulation of most of ER-stress related genes except *IRE1* similar to that observed in the 2D cultured HCSFs.

## Discussion

ROCK1 and 2 were initially identified throughout embryogenesis through adult tissues as ubiquitously expressed proteins^19,38^. Although the functional roles of both ROCK1 and 2 are thought to be similar, and siRNA knockdown study demonstrated that there is some difference in their functions. That is, in fibroblasts, ROCK1 and 2 act differently in the assembly of fibronectin matrices at the cell surface during actin cytoskeleton mediated extracellular matrix assembly^39,40^. Furthermore, although ROCK1 knockdown in keratinocytes induced a decrease in cell adhesion to fibronectin, the knockdown of ROCK2 promoted fibronectin adhesion^41,42^. These observations indicate that ROCK1 and 2 may have different roles and may well be localized in various tissues and organs. Only ROCK1 is cleaved by caspase-3 during apoptosis^43,44^ while smooth muscle-specific basic calponin is phosphorylated by ROCK2, but not ROCK1^45^. An expressed sequence tag (EST) analysis using the Tissue-specific Gene Expression and Regulation (TiGer) database^46^ demonstrated that ROCK1 and 2 are distributed similarly but, the distribution is substantially different in a few specific organs and/or tissues. ROCK1 is predominantly expressed in the thymus and blood, with little to no ROCK2 expression, while in contrast, ROCK2 is the most highly expressed of the two within cardiac and brain tissues in addition to the eye^19,47,48^.

It has been reported that ROCK1 and 2 are functionally identified as the major regulators of the cytoskeleton and are importantly involved in regulating cell movement through the formation of actin stress fibers and focal adhesion^49,50^. Both ROCK1 and 2 are also known to be involved in several corneal cell functions including epithelial differentiation^51^, proliferation^52^, cell adhesion^53^, cytoskeleton reorganization^54^, and cell–matrix interactions^55^. ROCK 1 and 2 are both activated in response to a wound and HB-EGF stimulation, and, the pan-ROCK inhibitor, Y-27632 stimulates wound healing mainly by modulating cell-ECM and cell–cell adhesion in HCECs^52,56^. Thus, it is thought that ROCKs regulate cell–cell adhesion mediated by E-cadherin and β-catenin, in addition to the formation and maintenance of barrier integrity^56^. However, the roles of each ROCK1 and 2 within human corneal cells, especially in HCSFs are not known with certainty. In the current study, to study such unidentified issues, we examined the drug induced effects of pan-ROCK-i, Rip and ROCK2-i, KD025, on the gene expressions of several ECM proteins, their modulators, TIMPS, MMPs, and several additional ER-stress related factors in 2D and 3D cultured HCSFs as well as on the physical properties of the 3D HCSFs spheroids (Table 1). Quite interestingly, both inhibitors induced diverse effects toward 2D and 3D HCSFs, and this led to the rational conclusion that ROCK1 and 2 may be involved in the spatial architecture of human corneal stroma indifferently manners.

**Table 1:** Summary of the effects of ROCK 1 and ROCK 2 on the physical properties of 3D human corneal stroma fibroblasts (HCSFs) spheroids and gene expression of 2D and 3D cultured HCSFs.

	1 mM Rip	10 mM Rip	1 mM KD025	10 mM KD025
Size	↑↑↑↑	↑↑↑↑	(–)	↑↑
Stiffness	(–)	↓↓	(–)	↑
***COL 1***				
2D	(–)	(–)	(–)	↓
3D	↓↓↓↓	↓↓↓↓	↓	↓↓↓↓
***COL 4***				
2D	(–)	↓	(–)	↑↑
3D	(–)	↓	(–)	(–)
***COL 6***				
2D	(–)	(–)	(–)	(–)
3D	(–)	(–)	↑↑↑	(–)
***FN***				
2D	(–)	↓↓↓↓	(–)	(–)
3D	↓↓	↓↓↓↓	↓↓	↓↓↓↓
***TIMP1***				
2D	(–)	(–)	(–)	(–)
3D	↓↓	↓↓↓↓	↓↓↓↓	(–)
***TIMP2***				
2D	(–)	(–)	(–)	↓↓↓↓
3D	↓↓↓	↓↓↓↓	(–)	(–)
***TIMP3***				
2D	(–)	↑↑	(–)	↓↓
3D	(–)	↓↓↓↓	(–)	↓↓↓↓
***TIMP4***				
2D	↓↓	(–)	↑↑↑↑	(–)
3D	(–)	↑↑↑↑	(–)	(–)
***MMP2***				
2D	(–)	(–)	(–)	↓↓↓↓
3D	↓	(–)	(–)	(–)
***MMP3***				
2D	(–)	(–)	↑↑↑↑	↑↑↑
3D	(––)	↑↑↑↑	(–)	(–)
***MMP9***				
2D	(–)	(–)	(–)	(–)
3D	(–)	(–)	↓↓↓↓	↓↓
***MMP14***				
2D	(–)	(–)	(–)	↓↓↓↓
3D	(–)	↑↑↑↑	(–)	(–)
***GRP 78***				
2D	(–)	(–)	(–)	(–)
3D	(–)	(–)	↓	↓↓↓↓
***GRP 94***				
2D	(–)	↓↓	↓↓↓	↓↓↓↓
3D	↑	(–)	↓↓↓↓	↓↓↓↓
***IRE 1***				
2D	(–)	(–)	(–)	↓
3D	(–)	(–)	(–)	(–)
***ATF 6***				
2D	(–)	(–)	(–)	↓↓
3D	(–)	(–)	↓↓↓	↓↓↓↓
***PERK***				
2D	(–)	(–)	(–)	↓↓
3D	(–)	(–)	↓↓↓↓	↓↓↓↓
***ATF 4***				
2D	(–)	↓↓↓↓	↓↓↓↓	↓↓↓↓
3D	(–)	(–)	↓↓↓↓	↓↓↓↓
***XBP 1***				
2D	(–)	(–)	(–)	↓↓
3D	(–)	↓	(–)	↓↓↓↓
***sXBP 1***				
2D	(–)	(–)	(–)	↑
3D	↓↓	↑↑	↓↓↓↓	↓↓↓↓
***CHOP***				
2D	(–)	(–)	(–)	↓↓↓↓
3D	(–)	↓	↓↓	↓↓↓↓

Rip: ripasudil, 2D: two-dimension culture, 3D: three-dimension culture, *COL*: collagen, *FN*: fibronectin, *TIMP*: tissue inhibitor of metalloproteinase, *MMP*: matrix metalloproteinase, protein kinase RNA-like endoplasmic reticulum kinase (PERK), activating transcription factor 6 (ATF6) and the inositol-requiring enzyme 1 (IRE1), and their downstream factors including the glucose regulator protein (GRP)78, GRP94, the X-box binding protein-1 (XBP1), spliced XBP1 (sXBP1) and CCAAT/enhancer-binding protein homologous protein (CHOP), (–): no significant change, ↑: significant increase (p < 0.05), ↑↑: significant increase (p < 0.01), ↑↑↑: significant increase (p < 0.005), ↓: significant decrease (p < 0.05), ↓↓: significant decrease (p < 0.01), ↓↓↓: significant decrease (p < 0.005).

In conclusion, the findings presented in this study indicate that 3D HCSFs spheroids represent a potential in vitro model for mimicking the 3D spatial architecture of the human corneal stroma, which is significantly influenced by ROCK isoforms. Therefore, this 3D HCSFs spheroid model has great potential for use as an approach for developing an understanding of the physiological and pathological aspects of the corneal stroma as well as its related diseases.

## Supplementary Information

Supplementary Figures.

Supplementary Table 1.
