# Supplementary material for: ROCK 1 and 2 affect the spatial architecture of 3D spheroids derived from human corneal stromal fibroblasts in different manners
Source: Sci Rep. 2022 May 6;12:7419. doi: 10.1038/s41598-022-11407-1 (PMC9076620; doi:10.1038/s41598-022-11407-1)
Supplement: Supplementary file 1 — Supplementary Figures. [file 41598_2022_11407_MOESM1_ESM.pdf]

ROCK1 and 2 affect the spatial architecture of 3D spheroids derived from human corneal stroma fibroblasts in different manners

Yosuke Ida[1], Araya Umetsu[1], Masato Furuhashi[2], Megumi Watanabe[1], Yuri Tsugeno[1], Soma Suzuki[1], Fumihito Hikage[1], Hiroshi Ohguro[1].

Departments of Ophthalmology[1] and Cardiovascular, Renal and Metabolic Medicine[2], Sapporo Medical University School of Medicine

Short title: Study of ROCK inhibitors on HCSFs by 3D culture

Key words: 3D spheroid culture, Rho-associated kinase (ROCK), ROCK inhibitor, human corneal stroma fibroblasts (HCSFs)

All correspondence should be addressed to Hiroshi Ohguro

Tel# 81-11-611-2111, Fax# 81-11-613-6575, e-mail: oogurο@sapmed.ac.jp

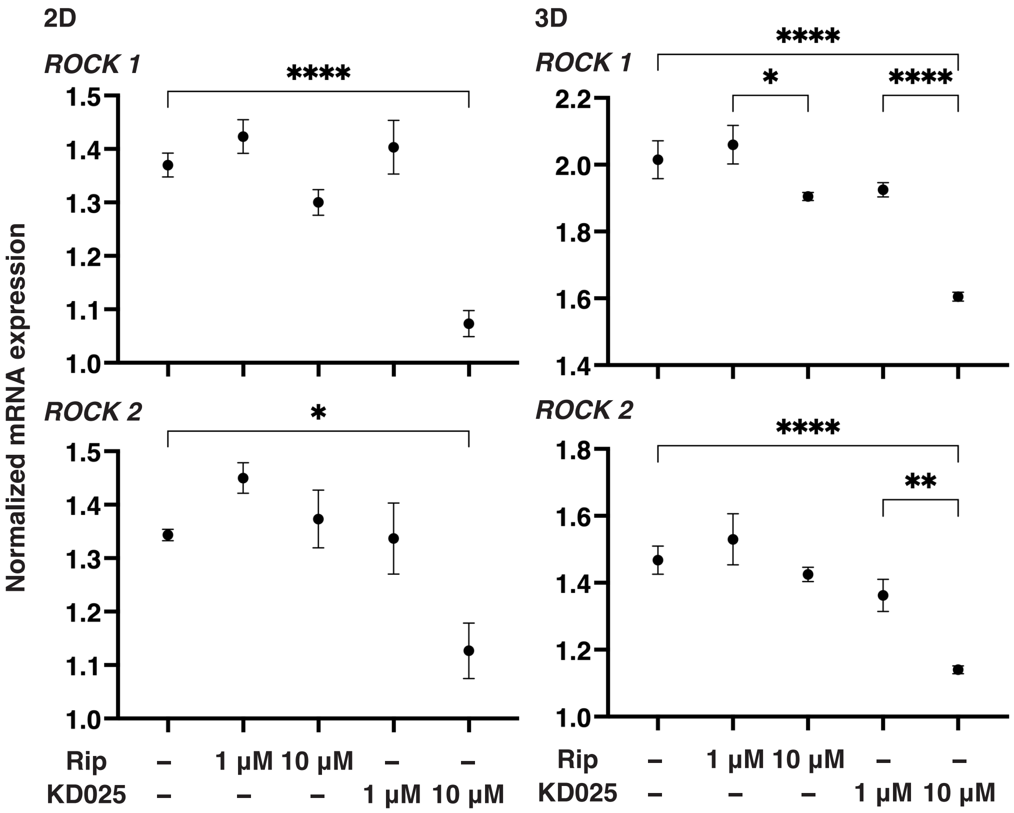

Supplemental Figure 1. mRNA expression of ROCK 1 and ROCK 2 in 2D and 3D human corneal stroma fibroblasts (HCSFs).

In the absence or presence of 1 µM or 10 µM ROCK-is, ripasudil (Rip) or KD025, 2D or 3D cultured HCSFs at Day 6 were subjected to qPCR analysis and the mRNA expression of ROCK 1 and ROCK 2 was estimated. All experiments were performed in duplicate using fresh preparations (2D; n=5, 3D; n=10-15, each). Data are presented as the arithmetic mean ± standard error of the mean (SEM). * $P<0.05$, ** $P<0.01$, **** $P<0.001$ (ANOVA followed by a Tukey's multiple comparison test).

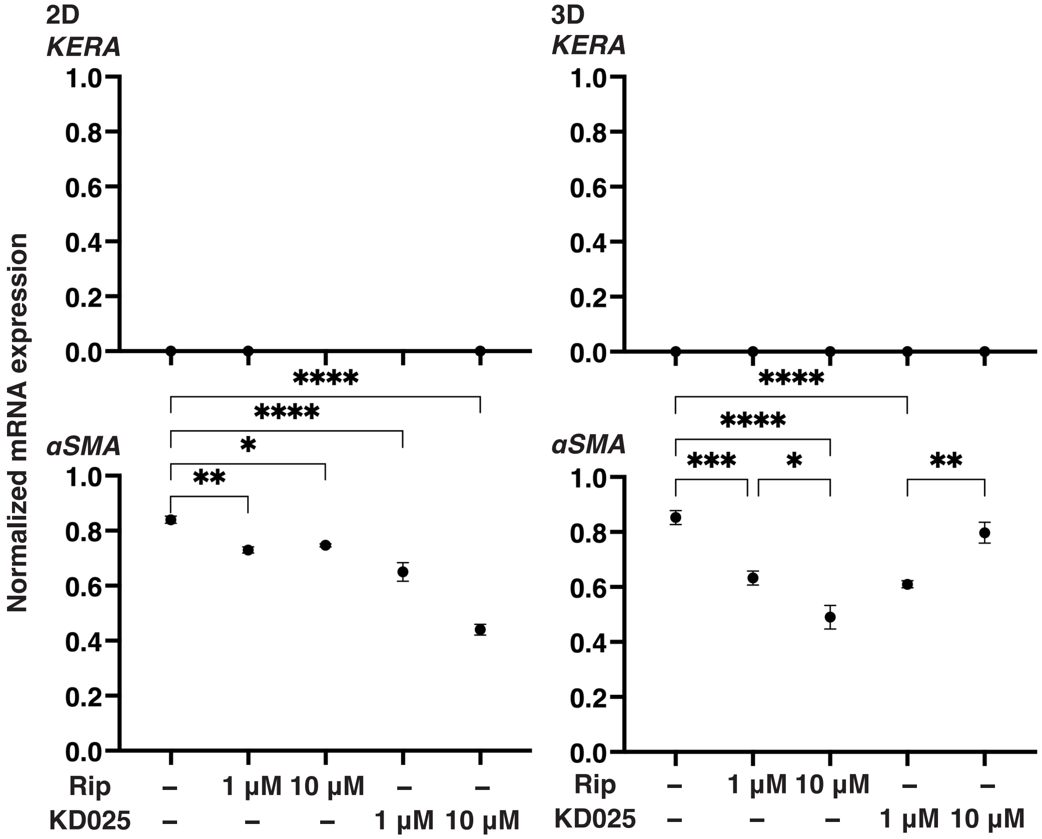

Supplemental Figure 2. mRNA expression of keratocan and αSMA in 2D and 3D human corneal stromal fibroblasts (HCSFs).

In the absence or presence of 1 µM or 10 µM ROCK-is, ripasudil (Rip), or KD025, 2D or 3D cultured HCSFs 6 were subjected to qPCR analysis at Day 6, and the mRNA expression of keratocan and αSMA estimated. All experiments were performed in duplicate using fresh preparations (2D; n=5, 3D; n=10-15, each). Data are presented as the arithmetic mean ± standard error of the mean (SEM). * $P<0.05$, ** $P<0.01$, *** $P<0.005$, **** $P<0.001$ (ANOVA followed by a Tukey's multiple comparison test).

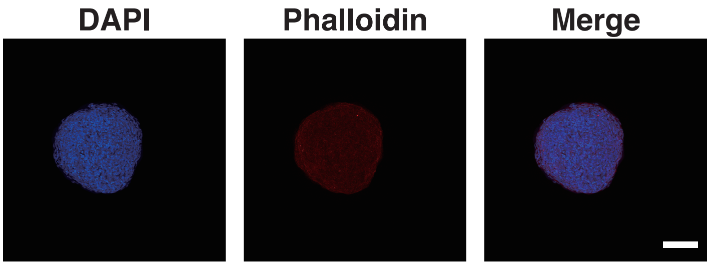

Supplemental Figure 3. Representative confocal images showing the DAPI and phalloidin in 3D human corneal stromal fibroblast (HCSFs) spheroids.

At Day 6, 3D cultures of spheroids of HCSFs were immune-stained with specific antibodies of DAPI by blue and phalloidin by red. Scale bar: 100 μm.
