# Supplementary material for: ROCK 1 and 2 affect the spatial architecture of 3D spheroids derived from human corneal stromal fibroblasts in different manners
Source: Sci Rep. 2022 May 6;12:7419. doi: 10.1038/s41598-022-11407-1 (PMC9076620; doi:10.1038/s41598-022-11407-1)
Supplement: Supplementary file 2 — Supplementary Table 1. [file 41598_2022_11407_MOESM2_ESM.pdf]

ROCK1 and 2 affect the spatial architecture of 3D spheroids derived from human corneal stroma fibroblasts in different manners

Yosuke Ida[1], Araya Umetsu[1], Masato Furuhashi[2], Megumi Watanabe[1], Yuri Tsugeno[1], Soma Suzuki[1], Fumihito Hikage[1], Hiroshi Ohguro[1].

Departments of Ophthalmology[1] and Cardiovascular, Renal and Metabolic Medicine[2], Sapporo Medical University School of Medicine

Short title: Study of ROCK inhibitors on HCSFs by 3D culture

Key words: 3D spheroid culture, Rho-associated kinase (ROCK), ROCK inhibitor, human corneal stroma fibroblasts (HCSFs)

All correspondence should be addressed to Hiroshi Ohguro

Tel# 81-11-611-2111, Fax# 81-11-613-6575, e-mail: ooguro@sapmed.ac.jp

		Sequence	Exon Location	RefSeqNumber
human RPLP0	Probe	5'-/56-FAM/CCCTGTCTT/ZEN/CCCTGGGCATCAC/3IABkFQ/-3'	2-3	NM_001002
	Forward	5'-TCGTCTTTAAACCCTGCGTG-3'		
	Reverse	5'-TGTCTGCTCCCACAATGAAAC-3'		
human COL1A1	Probe	5'-/56-FAM/TCGAGGGCC/ZEN/AAGACGAAGACATC/3IABkFQ/-3'	1-2	NM_000088
	Forward	5'-GACATGTTCAGCTTTGTGGAC-3'		
	Reverse	5'-TTCTGTACGCAGGTGATTGG-3'		
human COL4A1	Probe	5'-/56-FAM/TCATACAGA/ZEN/CTTGGCAGCGGCT/3IABkFQ/-3'	51-52	NM_001845
	Forward	5'-AGAGAGGAGCGAGATGTTCA-3'		
	Reverse	5'-TGAGTCAGGCTTCATTATGTTCT-3'		
human COL6A1	Forward	5'-CCTCGTGGACAAAGTCAAGT-3'	2-3	NM_001848
	Reverse	5'-GTGAGGCCTTGGATGATCTC-3'		
human FN1	Forward	5'-CGTCCTAAAGACTCCATGATCTG-3'	3-4	NM_212482
	Reverse	5'-ACCAATCTTGTAGGACTGACC-3'		
human TIMP1	Probe	5'-/56-FAM/TCAACCAGA/ZEN/CCACCTTATACCAGCG/3IABkFQ/-3'	2-4	NM_003254
	Forward	5'-GCTTGGAACCCTTTATACATCTTG-3'		
	Reverse	5'-CCTTCTGCAATTCCGACCT-3'		
human TIMP2	Probe	5'-/56-FAM/TCTCATTGC/ZEN/AGGAAAGGCCGAGG/3IABkFQ/-3'	3-4	NM_003255
	Forward	5'-TGTGGTTCAGGCTCTTCTTC-3'		
	Reverse	5'-GACGTTGGAGGAAAGAAGGA-3'		
human TIMP3	Probe	5'-/56-FAM/CCTCCTTTA/ZEN/CCAGCTTCTTCCCCAC/3IABkFQ/-3'	1-3	NM_000362
	Forward	5'-CGGTACATCTTCATCTGCTTGA-3'		
	Reverse	5'-CCTTCTGCAACTCCGACATC-3'		
human TIMP4	Probe	5'-/56-FAM/ACTGAGGAC/ZEN/CTGACCAGTCAAGAGA/3IABkFQ/-3'	4-5	NM_003256
	Forward	5'-GTTGCACAGATGGATGAAGAC-3'		
	Reverse	5'-GGTTTGAGAAAGTCAAGGATGTTC-3'		
human MMP2	Probe	5'-/56-FAM/TTCTGTCCC/ZEN/CATGAAGCCCTGTTC/3IABkFQ/-3'	6-7	NM_004530
	Forward	5'-GTGCAGCTGTCATAGGATGT-3'		
	Reverse	5'-TCCACCACCTACAACTTTGAG-3'		
human MMP3	Probe	5'-/56-FAM/TTCTGTCCC/ZEN/CATGAAGCCCTGTTC/3IABkFQ/-3'	6-7	NM_004530
	Forward	5'-GTGCAGCTGTCATAGGATGT-3'		
	Reverse	5'-TCCACCACCTACAACTTTGAG-3'		
human MMP9	Probe	5'-/56-FAM/CCAGGAGGA/ZEN/AAGGCGTGTGC/3IABkFQ/-3'	3-4	NM_004994
	Forward	5'-CGTCGAAATGGGCGTCT-3'		
	Reverse	5'-ACATCGTCATCCAGTTTGGTG-3'		
human MMP14	Probe	5'-/56-FAM/TTGTTCCTC/ZEN/AAAGTGCCTGTTTGCTC/3IABkFQ/-3'	1-1	NM_004995
	Forward	5'-CTTGAATTCCTAGACCGCTGT-3'		
	Reverse	5'-TTCGCCGACTAAGCAGAAG-3'		
human GRP78	Forward	5'-CATCACGCCGTCCTATGTCG-3'		NM_005347
	Reverse	5'-CGTCAAAGACCGTGTTCTCG-3'		
human GRP94	Forward	5'-CTGGGACTGGGAACTTATGAATG-3'		NM_003299
	Reverse	5'-TCCATATTCGTCAAACAGACCAC-3'		
human IRE1	Forward	5'-TTTGGAAGTACCAGCACAGTG-3'		NM_001433
	Reverse	5'-TGCCATCATTAGGATCTGGGA-3'		
human ATF6	Forward	5'-TCAGACAGTACCAACGCTTATGC-3'		NM_007348
	Reverse	5'-GTTGTACCACAGTAGGCTGAGA-3'		
human PERK	Forward	5'-ACGATGAGACAGAGTTGCGAC-3'		NM_004836
	Reverse	5'-AATCCCACTGCTTTTTACCATGA-3'		
human ATF4	Forward	5'-ATGACCGAAATGAGCTTCCTG-3'		NM_0016752
	Reverse	5'-CTGGAGAACCCATGAGGTTTG-3'		
human XBP1	Forward	5'-AGTAGCAGCTCAGACTGCCA-3'		NM_005080
	Reverse	5'-CCTGGTTCTCAACTACAAGGC-3'		
human sXBP1	Forward	5'-GGTCTGCTGAGTCCGCAGCAGG-3'		AB076384
	Reverse	5'-GGGCTTGGTATATATGTGG-3'		
human CHOP	Forward	5'-GGAGAACCAGGAAACGGAAAC-3'		NM_004083
	Reverse	5'-TCTCCTTCATGCGCTGCTTT-3'		
human KERA	Probe	5'-/56-FAM/TGTAATATG/ZEN/TCCCAGCCCATGC/3IABkFQ/-3'	2-3	NM_007035
	Forward	5'-GTTCCCCAATCAGTGCTC-3'		
	Reverse	5'-TGAGGTCCATAACTGAAGGAATC-3'		
human αSMA	Probe	5'-/56-FAM/AGACCCTGT/ZEN/TCCAGCCATCCTTC/3IABkFQ/-3'	1-1	NM_004995
	Forward	5'-CTGTTGTAGGTGGTTTCATGGA-3'		
	Reverse	5'-AGAGTTACGAGTTGCCTGATG-3'		

Supplemental Table 1 Primers for qPCR
